# Effects of Exosomes on Neurological Function Recovery for Ischemic Stroke in Pre-clinical Studies: A Meta-analysis

**DOI:** 10.3389/fncel.2020.593130

**Published:** 2020-11-26

**Authors:** Mudan Huang, Zhongqiu Hong, Chongjun Xiao, Lili Li, Lilin Chen, Shimei Cheng, Tingting Lei, Haiqing Zheng

**Affiliations:** Department of Rehabilitation Medicine, The Third Affiliated Hospital, Sun Yat-sen University, Guangzhou, China

**Keywords:** exosomes, animal models, ischemic stroke, cell-derived exosomes, meta-analysis

## Abstract

**Background:** Exosomes, especially stem cell-derived exosomes, have been widely studied in pre-clinical research of ischemic stroke. However, their pooled effects remain inconclusive.

**Methods:** Relevant literature concerning the effects of exosomes on neurological performance in a rodent model of ischemic stroke was identified via searching electronic databases, including PubMed, Embase, and Web of Science. The primary outcomes included neurological function scores (NFS) and infarct volume (IV), and the secondary outcomes were several pro-inflammatory factors and terminal deoxynucleotidyl transferase deoxyuridine triphosphate nick end labeling-positive cells. Subgroup analyses regarding several factors potentially influencing the effects of exosomes on NFS and IV were also conducted.

**Results:** We identified 21 experiments from 18 studies in the meta-analysis. Pooled analyses showed the positive and significant effects of exosomes on NFS (standardized mean difference −2.79; 95% confidence interval −3.81 to −1.76) and IV (standardized mean difference −3.16; 95% confidence interval −4.18 to −2.15). Our data revealed that the effects of exosomes on neurological outcomes in rodent stroke models might be related to routes of administration and exosomes sources. In addition, there was significant attenuation in pro-inflammatory factors, including interleukin-6, tumor necrosis factor-α and interleukin-1β, and terminal deoxynucleotidyl transferase deoxyuridine triphosphate nick end labeling-positive cells when undergoing exosomes treatment.

**Conclusion:** Cell-derived exosomes treatment demonstrated statistically significant improvements in structural and neurological function recovery in animal models of ischemic stroke. Our results also provide relatively robust evidence supporting cell-derived exosomes as a promising therapy to promote neurological recovery in stroke individuals.

## Introduction

Ischemic stroke continues to be a leading cause of adult death and permanent disability throughout the world (Chen L. et al., 2016). In most instances, ischemic stroke is caused by blood-vessel occlusion, most commonly due to an embolus or local thrombosis (Dirnagl et al., 1999). Given the limitation of available therapeutic options, patients still retain long-term dysfunction after stroke, although they have received recombinant tissue plasminogen activator or endovascular intervention (Schwamm et al., 2013; Nogueira et al., 2018).

To replace the loss of the functional neurons, stem cell transplantation represents a promising treatment option (Boltze et al., 2014). Several studies have identified that transplantation of mesenchymal stem cells (MSCs) not only facilitated functional recovery in rodents after stroke but also improved neurological outcomes for post-stroke survivors (Liu et al., 2011; Levy et al., 2019). However, only a small percentage of MSCs engrafted into the ischemic hemisphere exerted neuroprotective effects (Acosta et al., 2015; Gervois et al., 2016). In addition, cell-based therapy was found with several potential risks and limitations, such as tumor formation, occlusion in the microvasculature, and weak capacity to cross the blood–brain barrier (Lukomska et al., 2019).

Emerging studies demonstrated that exosomes released from MSCs are the major biological principle underlying the therapeutic benefits of MSC transplantation (Xin et al., 2013b; Doeppner et al., 2015). Exosomes are nanosized vesicles with a size of 40 to 150 nm and secreted by different cell types. They play a pivotal role in mediating intercellular communication by delivering a variety of functional biomolecules, including messenger RNAs, microRNAs (miRNAs), long non-coding RNA, proteins, and lipids, to recipient cells (Raposo and Stoorvogel, 2013; Tkach and Théry, 2016). Until now, exosomal miRNAs have been investigated more fully than other cargo of exosomes in the light of their functional significance. Indeed, increasing data showed that exosomal miRNAs improved neurological function and promoted neurovascular remodeling in the ischemic brain (Xin et al., 2013b, 2017a). Furthermore, nanometer-sized exosomes are admitted to cross the blood–brain barrier and prevent tumor formation. Therefore, cell-derived exosomes are expected to become an efficient and safe treatment for ischemic stroke.

A considerable number of animal studies have been performed to explore the efficacy of exosomes on an ischemic stroke model, but they have various cell origins, administration routes, injection doses, and timepoints of intervention and evaluation (Song et al., 2019; Zhao et al., 2020). However, there are a few clinical trials concerning cell-derived exosomes in the treatment of ischemic stroke, and most of them are in the initial recruitment stage of the trials. To characterize and quantify the pre-clinical evidence and provide a vital theoretical basis for clinical application, we therefore aimed to conduct this meta-analysis of animal studies.

## Materials and Methods

The protocol was established according to the Preferred Reporting Items for Systematic Review and Meta-Analysis guidelines (Moher et al., 2009).

### Search Strategy

We conducted a literature search in the electronic database, including PubMed, Embase, and Web of Science. The search strategy was as follows: (“exosomes” or “exosome” or “exosomal” or “secretome”) and (“brain ischemia” or “cerebral ischemia” or “ischemia stroke” or “brain infarct” or “cerebral infarct” or “middle cerebral artery occlusion” or “MCAO”). All of the search strategies were performed from the initiation to June 2020, and the literature was published in English. The references of selected articles were further searched by hand to obtain additional citations.

### Study Selection

Two authors (MH and ZH) independently abstracted all data from any eligible publication, according to a standard protocol. Discrepancies were resolved by discussion with the third reviewer (HZ). The inclusion criteria for this research were (1) population-rodent models with ischemic stroke (transient or permanent); (2) intervention unmodified cell-derived exosomes; (3) comparison-vehicle, phosphate-buffered saline, or positive control drug or no treatment; and (4) outcome measure—the primary outcomes were neurological function score (NFS) and infarct volume (IV). The secondary outcomes were the percentage of apoptotic cells, levels of interleukin (IL)-1β, IL-6, and tumor necrosis factor (TNF)-α. The exclusion criteria were (1) ischemic stroke was not conducted on rodent models; (2) artificially modified cell-derived exosomes; (3) repeated data or insufficient information; (4) the intervention was a combination of exosomes and another drug; and (5) clinical articles, case reports, commentary, reviews, conference abstracts, correspondence, expert opinion, and *in vitro* studies.

### Data Extraction

The following data extraction from the included studies was conducted independently by two reviewers (MH and ZH). The following details were collected: (1) name of the first author and the publication year; (2) country or region; (3) animal sex and species; (4) number of animals in the study, control group, and exosomes treatment group; (5) comorbid status of animals; (6) method of an ischemic stroke model; (7) occlusion time of animal with ischemic stroke; (8) treatment time; (9) origins of exosome; (10) administration route of exosome; (11) administration dose of exosome; (12) measurement time; and (13) outcome measures. For included studies without available numerical values in the text, we contacted with authors twice by email and performed the WebPlotDigitizer software (https://automeris.io/WebPlotDigitizer/) to extract data from the figures or graphs (Burda et al., 2017).

### Quality Assessment

The methodological quality of each eligible studies was assessed independently by two investigators (MH and ZH) via using the 10-item Collaborative Approach to Meta-Analysis and Review of Animal Data from Experimental Studies quality checklist and detailed items as follows: *A*, publication in a peer-reviewed journal; *B*, statement of temperature control; ***C***, randomization to treatment or control group; *D*, blinding of the ischemic model establishment; *E*, blinding of outcome assessment; *F*, anesthetic without obvious intrinsic vascular protection activity; *G*, an appropriate animal model such as advanced age, diabetic, or hypertensive; *H*, estimation of the sample size; *I*, compliance with animal welfare regulations; and *J*, declaration of potential conflicts of interest (Zhao et al., 2020).

### Effect Sizes Estimation

The effect size was calculated using standardized mean difference (SMD) with 95% confidence intervals (95% CIs) for continuous outcomes because a single outcome measure was assessed and reported across trials using different measurement tools. For quantitative synthesis, the pooled effect estimation was calculated by comparing the change from baseline with the endpoint of the study between the intervention group and the control group.

The primary outcome was served to assess functional and structural recovery, which included NFSs and IV. If more than one measure for NFS were used in an individual trial, a modified neurological severity score was considered as a priority outcome measure because it is more appropriate to reflect neurological impairment; otherwise, neurological severity score and various behavioral tests (adhesive removal test, foot-fault test). We choose the 2,3,5-triphenyl tetrazolium chloride method as IV assessment after considering MRI and cresyl violet staining. Several pro-inflammatory factors, such as IL-6, TNF-α, and IL-1β, and the percentage of terminal deoxynucleotidyl transferase deoxyuridine triphosphate nick end labeling-positive cells, were detected as the secondary outcomes. Besides, several subgroup analyses were conducted to investigate whether study characteristics (administration routes, type of ischemia, exosomes sources, intervention time, and different species) had the effects on exosomes in improving neurological performance via using random-effects models. The authors were contacted twice by email for original data if the published study data were insufficient for data analyses.

### Statistical Analysis

All statistical analyses were performed by Review Manager 5.3 (The Cochrane Collaboration, Oxford, UK) and Stata version 14.0 (Stata Corp, College Station, TX, USA). We used the SMD with the 95% CI to record the continuous outcomes. Statistical heterogeneity was analyzed by using the I-square test. A random-effects model test was performed when heterogeneity was significant (*I*^2^ > 50%). Subgroup and sensitivity were conducted to investigate the origin of heterogeneity. A value of *p* < 0.05 was considered statistically significant.

## Results

### Search Results and Study Characteristics

**Figure 1:**
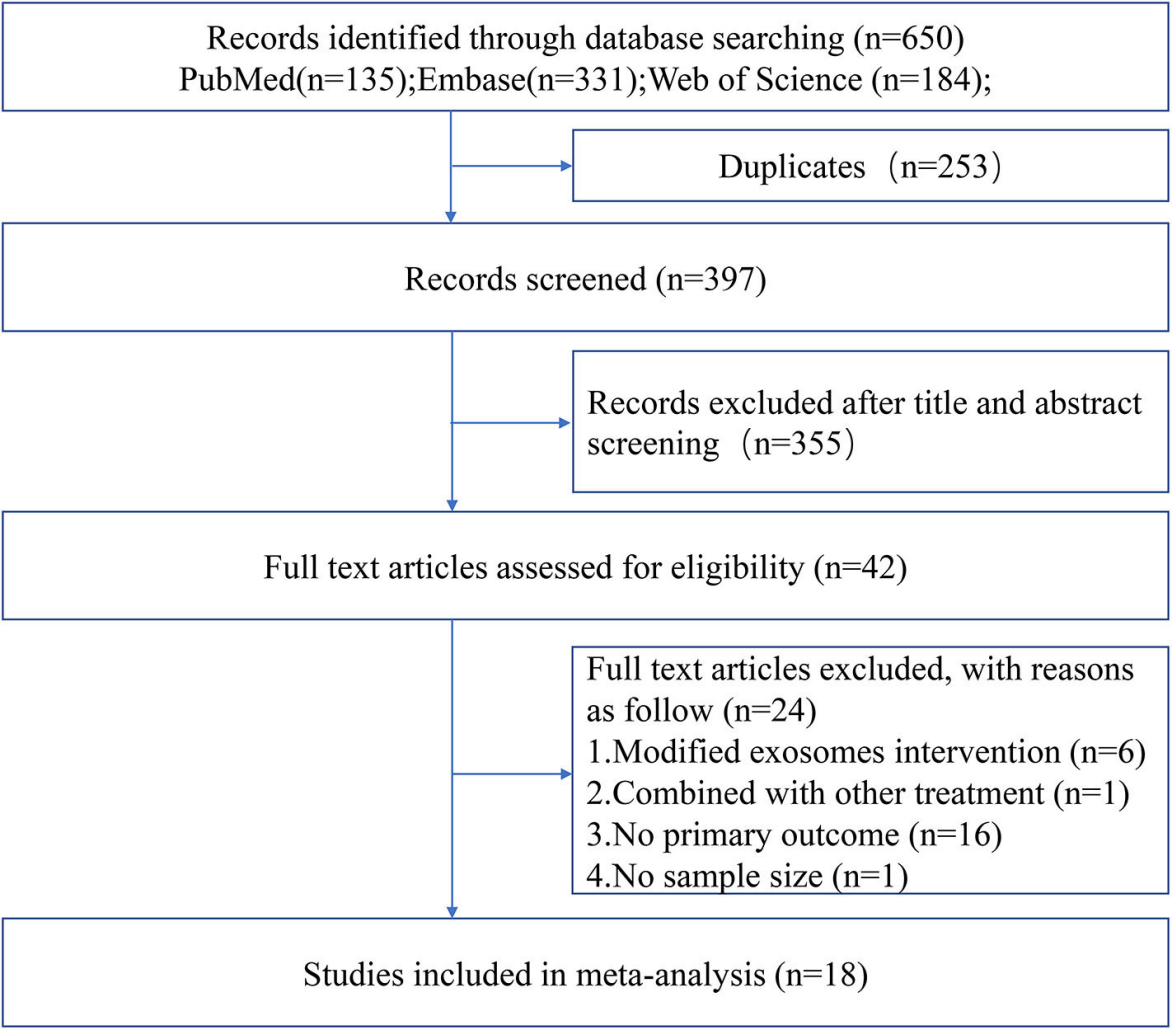
Flow chart of the study selection.

The process of study selection is outlined in Figure 1. A total of 18 studies with 21 comparisons satisfied the inclusion criteria (Xin et al., 2013a, 2017a,b; Chen K. H. et al., 2016; Jiang et al., 2018; Deng et al., 2019; Li et al., 2019; Nalamolu et al., 2019a,b; Pei et al., 2019; Song et al., 2019; Sun et al., 2019; Venkat et al., 2019; Zheng et al., 2019; Li G. et al., 2020; Ling et al., 2020; Safakheil and Safakheil, 2020; Zhao et al., 2020). The characteristics of the included studies are presented in Table 1. Moreover, the Preferred Reporting Items for Systematic Review and Meta-Analysis flow diagram was showed in Supplementary Table 1. All these studies were published between 2013 and 2020, and 367 animals were included in this meta-analysis. Among the included studies, six of them used bone marrow mesenchymal stromal cells—exosome (Xin et al., 2013a, 2017a,b; Deng et al., 2019; Safakheil and Safakheil, 2020; Zhao et al., 2020), one adopted adipose-derived mesenchymal stem cells—exosome (Chen K. H. et al., 2016), one applied human umbilical cord mesenchymal stem cells—exosome (Li G. et al., 2020), one used human umbilical cord blood mesenchymal stromal cells—exosome (Nalamolu et al., 2019b), one used adipose-derived stem cells—exosome(Jiang et al., 2018), one used neural stem cells—exosome (Sun et al., 2019), one used human urine-derived stem cells—exosome (Ling et al., 2020), one used mouse brain endothelial cells—exosome (Venkat et al., 2019), one used macrophages—exosome (Zheng et al., 2019), one used microglia—exosome, and one used astrocytes—exosome (Pei et al., 2019; Song et al., 2019), and the two remaining used another origin of exosomes (Li et al., 2019; Nalamolu et al., 2019a).

**Table 1:** Characteristics of included studies.

**References**	**Country or region**	**Gender and species**	**Animal number**	**Comorbid status**	**Occlusion method**	**Occlusion time**	**Therapy time**	**Exosomes source**	**Administration method**	**Therapy dose**	**Measure time**	**Outcome measure**
Xin et al. (2017a)	USA	Male Wistar rats	T:24 C:8 E:8	Healthy	Filament insertion	2 h	24 h after operation	BMSCs	Intravenous administration	100 μg	1, 3, 7, 14, 21, and 28 days after stroke (C:8 and E:8 /timepoint)	1. Behavioral tests (mNSS, Foot-fault test); 2. Axonal density, phosphorylated NF-H, synaptophysin; 3. Neurite branching, spine density; 4. NeuN/Brdu, NG2/Brdu, MBP/Brdu
Zhao et al. (2020)	China	Male SD rats	T:24 C:8 E:8	Healthy	Filament insertion	90 min	2 h after operation	BMSCs	Tail vein	120 μg	1, 3, and 7 days after stroke (C:8 and E:8 /timepoint)	1. Behavioral tests (NSS, shuttle box test); 2. CD206 and CD86; 3. NO, IL-1β, TNF-α, IL-12, IL-10, TGF-β, BDNF, GDNF (ELISA, brain tissue).
Song et al. (2019)	China	Male ICR mice	T:48 C:8 E:8	Healthy	Filament insertion	1 h	Immediately after operation	M2 microglia	Tail vein	100μg/day,3 days	3 days after stroke (C:8 and E:8)	1. Infarct volumes (Cresyl Violet Staining); 2. Behavioral tests (mNSS); 3. Apoptosis (TUNEL)
Pei et al. (2019)	China	Male C57BL/6 mice	T:60 C:10 E:10	Healthy	Filament insertion	Permanent	60 min after operation	Astrocytes	Tail vein	80 μg	3 days after stroke (C:10 and E:10)	1. Infarct volumes (TTC); 2. Apoptosis (TUNEL); 3. GFAP, Iba-1; 4. Beclin-1, LC3-I/II, P62; 5. TNF-α, IL-6. IL-1β (ELISA, brain tissue).
Safakheil and Safakheil (2020)	Iran	MaleWistar rats	T:60 C:12 E:12	Healthy	Filament insertion	60 min	24 h after operation	BMSCs	Stereotaxic (brain cortex)	100 μg	24 h,7 days after stroke (C:12 and E:12 /timepoint)	1. Behavioral tests (EBST, Garcia); 2. Infarct volume (TTC); 3. Dead cells (Cresyl Violet Staining); 4. NLRP1, NLRP3; 5. GFAP-positive cells; 6.MDA, SOD
Deng et al. (2019)	China	NRC57BL/6 mice	T:40 C:10 E:10	Healthy	Filament insertion	30 min	NR	BMSCs	NR	NR	4 weeks after treatment (C:10 and E:10)	1. Infarct volumes (TTC); 2. Apoptosis (TUNEL); 3. LDH, cleaved caspase-3, Bax, Bcl-2; 4. IL-6, IL-1β, TNF-α (WB, brain tissue).
Jiang et al. (2018)	China	Male SD rats	T:24 C:6 E:6	Healthy	Filament insertion	Permanent	Immediately after operation	ADSCs	Tail vein	80 μg	3 h, 72 h after stroke (C:6 and E:6 /timepoint)	1. Infarct volumes (TTC); 2. Apoptosis (TUNEL); 3. TNF-α, IL-6, IL-4, IL-10 (ELISA, serum). 4. CD206, iNOS, Beclin-1, Atg5, LC3
Zheng et al. (2019)	China	Male SD rats	T:18 C:6 E:6	Healthy	Filament insertion	2 h	Immediately after operation	Macrophages	Tail vein	2 mg	6 h (C:6 and E:6), 24 h after stroke (C:3 andE:3)	1. Infarct volumes (TTC); 2. Behavioral tests (Zea-Longa, Ludmila Belayev); 3. CD 80, CD206, NeuN- positive cells; 4. IL-6, TNF-α, NF-κB p65 (WB, brain tissue).
Li et al. (2019)	China	Male C57BL/6 mice	T:42-48 C:14-16 E:14-16	Healthy	Bipolar electrocoat-gulation	Permanent	Immediately after operation	RIPC mice plasma	Tail vein	10 μg/day,14 days	24 h (C:14-16 and E:14-16) and 3, 7, 14, 21, 28 days after stroke (C:8-10 and E:8-10 /timepoint)	1. Infarct volumes (TTC); 2. Behavioral tests (rotarod test, adhesive removal test); 3. HIF-1α
Li et al. (2019)	China	Male C57BL/6 mice	T:42-48 C:14-16 E:14-16	Healthy	Bipolar electrocoat-gulation	Permanent	Immediately after operation	Non-RIPC mice plasma	Tail vein	10 μg/day,14 days	24 h (C:14-16 and E:14-16) and 3, 7, 14, 21, and 28 days after stroke (C:8-10 and E:8-10 /timepoint)	1. Infarct volumes (TTC); 2. Behavioral tests (rotarod test, adhesive removal test); 3. HIF-1α
Sun et al. (2019)	United States	Male CB57/B6 mice	T:30 C:15 E:15	Healthy	Filament insertion	1 h	2 h after operation	NSCs	Internal jugular vein	10 μg	24 h, 4 days after stroke (C:15 and E:15 /timepoint)	1. Infarct volumes (TTC); 2. Behavioral tests (neurologic deficit score)
Nalamolu et al. (2019b)	USA	Male SD rats	T:30 C:15 E:15	Healthy	Filament insertion	2 h	Immediately after operation	HUCB-MSCs	Tail vein	150 μg	1 (C:15 and E:15), 3, 5, and 7 days after stroke (C:9 and E:9 /timepoint)	1. Infarct volumes (TTC); 2. Brain swelling; 3. Behavioral tests (mNSS, adhesive removal test, Beam-walking, Rotarod); 4. Body weight changes and mortality
Nalamolu et al. (2019a)	USA	Male SD rats	T:36 C:12 E:12	Healthy	Filament insertion	2 h	Immediately after operation	Cocultures of normal and OGD-induced HUCB-MSCs	Tail vein	150 μg	1 (C:12 and E:12), 3 (C:10 and E:10), 5, and 7 days after stroke (C:8 and E:8 /timepoint)	1. Infarct volumes (TTC); 2. Brain swelling; 3. Behavioral tests (mNSS, adhesive removal test, Beam-walking); 4. Body weight changes and mortality
Nalamolu et al. (2019a)	USA	Male SD rats	T:36 C:12 E:12	Healthy	Filament insertion	2 h	Immediately after operation	OGD-induced HUCB-MSCs	Tail vein	150 μg	1 (C:12 and E:12), 3 (C:10 and E:10), 5, and 7 days after stroke (C:8 and E:8 /timepoint)	1. Behavioral tests (mNSS, adhesive removal test, Beam-walking); 2. Body weight changes and mortality
Li G. et al. (2020)	China	Wild Balc/C mice	T:124 C:20 E:20	Healthy	Filament insertion	NR	Immediately after operation	hUCMSCs	Tail vein	5 μg	24 h after stroke (C:20 and E:20)	1. Infarct volumes (TTC); 2. Brain edema; 3. Behavioral tests (Longa)
Li S. et al. (2020)	China	Wild Balc/C mice	T:124 C:20 E:20	Healthy	Filament insertion	NR	Immediately after operation	hUCMSCs	Tail vein	50 μg	24 h after stroke (C:20 and E:20)	1. Infarct volumes (TTC); 2. Brain edema; 3. Behavioral tests (Longa); 4. TNF-?, IL-6, and CCL-2 (ELISA, brain tissue). 5. iNOS, Arg1, CD38^+^, CD206^−^
Chen K. H. et al. (2016)	Taiwan	Male SD rats	T:60 C:12 E:12	Healthy	Filament insertion	50 min	3 h after operation	Xenogenic ADMSCs	Intravenous administration	100 μg	1, 3, 14, 28, and 60 days after stroke (C:12 and E:12/timepoint)	1. Infarct volumes (MRI); 2. Behavioral tests (Corner test); 3. MMP-9, IL-1β, TNF-α, RANTES, PAI-1, NF-κB and iNOS (WB, brain tissue). 4. NOX-1, NOX-2 and oxidized protein; 5. Cleaved caspase 3 and cleaved PARP, Smad3, TGF-β, Smad1/5 and BMP-2; 6. γ-H2AX, cytosolic cytochrome C, CD31, eNOS, VEGF, CXCR4; 7. CD11, CD68, XRCC1/CD90, p53BP1/CD90, GFAP, AQP4
Ling et al. (2020)	China	Male SD rats	T:40 C:10 E:10	Healthy	Filament insertion	2 h	4 h after operation	hUSCs	Intravenous administration	1 × 10^11^ particle	1, 2, 3, 7, 14, 21, and 28 days after stroke (C:10 and E:10/timepoint)	1. Infarct volumes (MRI/cresyl violet staining); 2. Behavioral tests (mNSS, foot-fault test); 3. EdU^+^/Nestin^+^, EdU^+^/Sox2^+^.
Xin et al. (2013a)	USA	Male Wistar rats	T:12 C:6 E:6	Healthy	Filament insertion	2 h	24 h after operation	BMSCs	Tail vein	100 μg	1, 3, 7, 14, 21, and 28 days after stroke (C:6 and E:6/timepoint)	1. Infarct volumes (HE); 2. Behavioral tests (mNSS, Foot-fault test); 3. Axonal density, synaptophysin; 4. DCX/BrdU and vWF/Brdu
Venkat et al. (2019)	USA	male BKS.Cg-m+/+Lepr^db^ /J mice	T:25 C:7 E:6	T2DM	Photothro-mbotic	Permanent	3 days after operation	Mouse brain endothelial cells	Intravenous administration	3 × 10^10^	1, 7, 14, 21, 25, 26, 27, and 28 days after stroke (C:7 and E:6/timepoint)	1. Infarct volumes (HE); 2. Behavioral tests (adhesive removal test, novel odor recognition test); 3. Axon and Myelin Density, Vascular Density, Arterial Diameter, Vessel Patency; 4. ED1 and CD163
Xin et al. (2017b)	USA	Male Wistar rats	T:24 C:6 E:6	Healthy	Filament insertion	2 h	24 h after operation	BMSCs	Intra-arterial administration	100 μg	1, 3, 7, 14, 21, and 28 days after stroke (C:6 and E:6/timepoint)	1. Behavioral tests (mNSS, Foot-fault test); 2. Axonal density, synaptophysin, SMI-31

*TTC, 2,3,5-triphenyl tetrazolium chloride; BMSCs, bone marrow mesenchymal stem cells; EBST, elevated body swing test; LDH, lactate dehydrogenase; NR, not reported; SD, Sprague-Dawley; C, control group; T, treatment group; ADSCs, adipose-derived stem cells; ADMSCs, adipose-derived mesenchymal stem cells; RIPC, remote ischemic pre-conditioning; NSCs, neural stem cells, HUCB-MSCs human umbilical cord blood-mesenchymal stem cells; mNSS, modified neurological severity scores; OGD, oxygen–glucose-deprived, hUCMSCs, human umbilical cord mesenchymal stem cells; hUSCs, human urine-derived stem cells; HE, hematoxylin and eosin; vWF, von Willebrand factor; T2DM, type 2 diabetes mellitus; BDNF, brain-derived neurotrophic factor; GDNF, glial cell-derived neurotrophic factor; TGF, transforming growth factor; ELISA, enzyme-linked immunosorbent assay; WB Western blot analysis; T, total animal in this study; C, control group; E, exosomes treatment group; TUNEL, terminal deoxynucleotidyl transferase deoxyuridine triphosphate nick end labeling*.

The ischemic stroke model was established with a unifilar nylon suture (Xin et al., 2013a, 2017a,b; Chen K. H. et al., 2016; Jiang et al., 2018; Deng et al., 2019; Nalamolu et al., 2019a,b; Pei et al., 2019; Song et al., 2019; Sun et al., 2019; Zheng et al., 2019; Li G. et al., 2020; Ling et al., 2020; Safakheil and Safakheil, 2020; Zhao et al., 2020), bipolar electrocoagulation forceps (Li et al., 2019), or thrombus (Venkat et al., 2019). A total of 13 studies used the transient ischemic stroke model (Xin et al., 2013a, 2017a,b; Chen K. H. et al., 2016; Deng et al., 2019; Nalamolu et al., 2019a,b; Song et al., 2019; Sun et al., 2019; Zheng et al., 2019; Ling et al., 2020; Safakheil and Safakheil, 2020; Zhao et al., 2020), and four studies used the permanent model (Jiang et al., 2018; Li et al., 2019; Pei et al., 2019; Venkat et al., 2019). Most studies chose to perform exosomes injection immediately after the injury model was established (Jiang et al., 2018; Li et al., 2019; Nalamolu et al., 2019a,b; Song et al., 2019; Zheng et al., 2019; Li G. et al., 2020), and several studies reported the same dose of exosomes (100 μg) injected intravenously (Xin et al., 2013a, 2017a,b; Chen K. H. et al., 2016; Safakheil and Safakheil, 2020). A variety of miRNA types have been reported in exosomes, such as miR-17-92 (Xin et al., 2017a), miR-126 (Venkat et al., 2019), miR-124 (Song et al., 2019), miR-26a (Ling et al., 2020), miR-26b-5p (Li G. et al., 2020), miR-30d-5p (Jiang et al., 2018), and miR-138-5p (Deng et al., 2019).

### Quality Assessment

The median quality score across the 18 studies was 5.5, and the range was from 4 to 7. The most widely met criteria included peer-reviewed publications (100% of studies), appropriate animal model (100% of studies), statement of compliance with animal welfare regulations (100% of studies), and statement of potential conflict of interests (100% of studies). Moreover, 72.22% of the included studies were randomly divided into the treatment and control groups, whereas the randomization was not mentioned in the remaining studies (27.78%). However, the calculation of sample size, the blinded induction of stroke model, and the blinded assessment of the experimental outcome had not been reported in all included studies. Further details of the study quality score are presented in Table 2.

**Table 2:** Quality assessment of eligible studies.

**References**	**A**	**B**	**C**	**D**	**E**	**F**	**G**	**H**	**I**	**J**	**Total**
Xin et al. (2017a)	**√**		**√**			**√**	**√**		**√**	**√**	6
Zhao et al. (2020)	**√**	**√**				**√**	**√**		**√**	**√**	6
Song et al. (2019)	**√**		**√**			**√**	**√**		**√**	**√**	6
Pei et al. (2019)	**√**	**√**	**√**			**√**	**√**		**√**	**√**	7
Safakheil and Safakheil (2020)	**√**	**√**				**√**	**√**		**√**	**√**	6
Deng et al. (2019)	**√**		**√**			**√**	**√**		**√**	**√**	6
Jiang et al. (2018)	**√**	**√**				**√**	**√**		**√**	**√**	6
Zheng et al. (2019)	**√**		**√**			**√**	**√**		**√**	**√**	6
Li et al. (2019)	**√**		**√**				**√**		**√**	**√**	5
Sun et al. (2019)	**√**		**√**			**√**	**√**		**√**	**√**	6
Nalamolu et al. (2019b)	**√**	**√**	**√**			**√**	**√**		**√**	**√**	7
Nalamolu et al. (2019a)	**√**		**√**				**√**		**√**	**√**	5
Li G. et al. (2020)	**√**	**√**	**√**				**√**		**√**	**√**	6
Chen K. H. et al. (2016)	**√**	**√**				**√**	**√**		**√**	**√**	6
Ling et al. (2020)	**√**		**√**				**√**		**√**	**√**	5
Xin et al. (2013a)	**√**						**√**		**√**	**√**	4
Venkat et al. (2019)	**√**		**√**			**√**	**√**		**√**	**√**	6
Xin et al. (2017b)	**√**		**√**				**√**		**√**	**√**	5

*A peer-reviewed journal; B temperature control; C animals were randomly allocated; D blind established model; E blinded outcome assessment; F use of anesthetic without significant intrinsic vascular protection activity; G appropriate animal model (diabetic, advanced age, or hypertensive); H calculation of sample size; I statement of compliance with animal welfare regulations; J statement of potential conflict of interests*.

### Primary Outcomes

#### Neurological Function Scores

**Figure 2:**
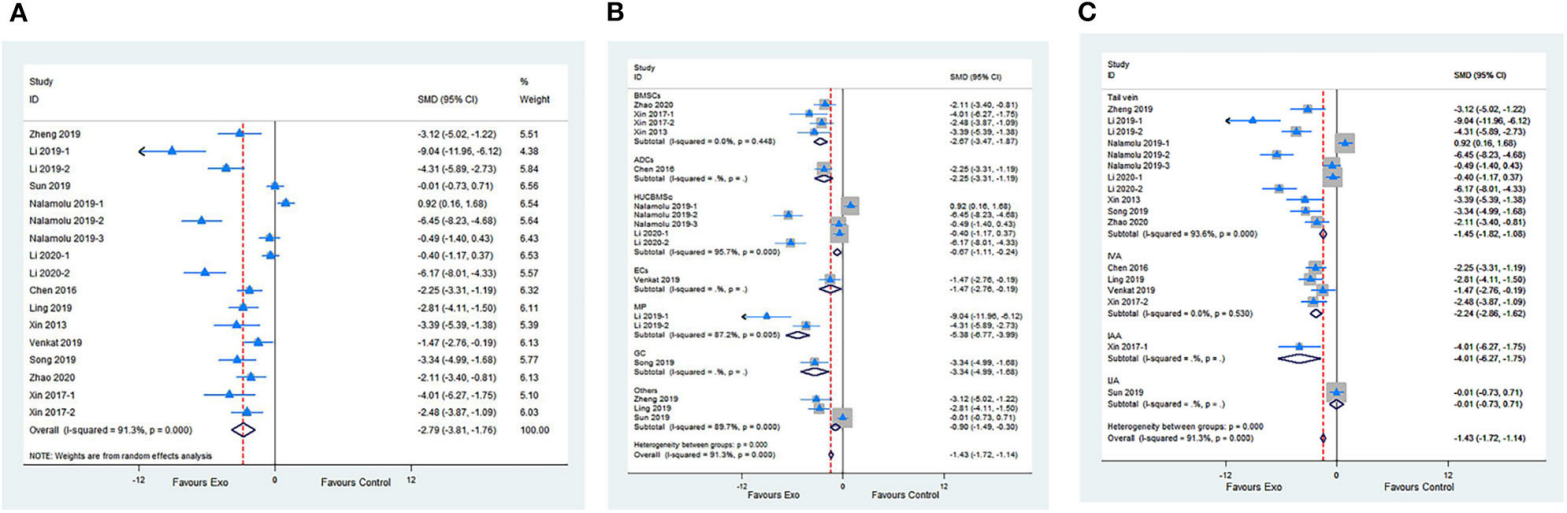
**(A)** Forest plot shows the efficacy of exosomes in improving the neurological function in the ischemic stroke model. **(B)** Forest plot shows the efficacy of exosomes derived from various cells in improving the neurological function in the ischemic stroke model. **(C)** Forest plot shows the efficacy of exosomes via different administrational routes in improving the neurological function in the ischemic stroke model.

Fourteen studies with 17 comparisons reported the NFSs. The pooled analysis showed that exosomes can significantly improve the neurological function compared with the control (SMD −2.79; 95% CI −3.81 to −1.76; *p* < 0.001; *I*^2^ = 91.3%; Figure 2A). The subgroup analysis showed that all cell-derived exosomes are effective in improving neurological function (Figure 2B). Moreover, the subgroup analysis showed that all administrational routes help improve the neurological function, except for internal jugular vein administration (Figure 2C). In addition, the subgroup analysis presented that immediate or delayed treatment with cell-derived exosomes could improve the neural recovery after transient or permanent stroke (Supplementary Figures 1A,B; Supplementary Table 2). The sensitivity analysis showed that none of the single studies significantly influenced the result (Supplementary Figure 1C).

#### Infarct Volume

**Figure 3:**
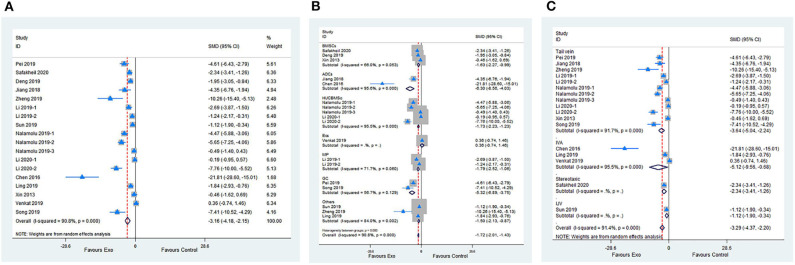
**(A)** Forest plot shows the efficacy of exosomes in reducing infarct volume in the ischemic stroke model. **(B)** Forest plot shows the efficacy of exosomes derived from various cells in reducing infarct volume in the ischemic stroke model. **(C)** Forest plot shows the efficacy of exosomes via different administrational routes in reducing infarct volume in the ischemic stroke model.

Meta-analysis of 15 studies with 18 comparisons showed significant effects of exosomes for reducing IV compared with control groups (SMD −3.16; 95% CI −4.18 to −2.15; *p* < 0.001; *I*^2^ = 90.8%; Figure 3A). The subgroup analysis presented that all cell-derived exosomes benefit from reducing IV except for mouse brain endothelial cells (Figure 3B). Moreover, the subgroup analysis showed that all administrational routes effectively reduce IV (Figure 3C). In addition, the subgroup analysis indicated that immediate or delayed treatment with cell-derived exosomes reduced IV after transient or permanent stroke (Supplementary Figures 2A,B; Supplementary Table 2). None of the single studies significantly influenced the result via sensitivity analysis (Supplementary Figure 2C).

### Secondary Outcomes

**Figure 4:**
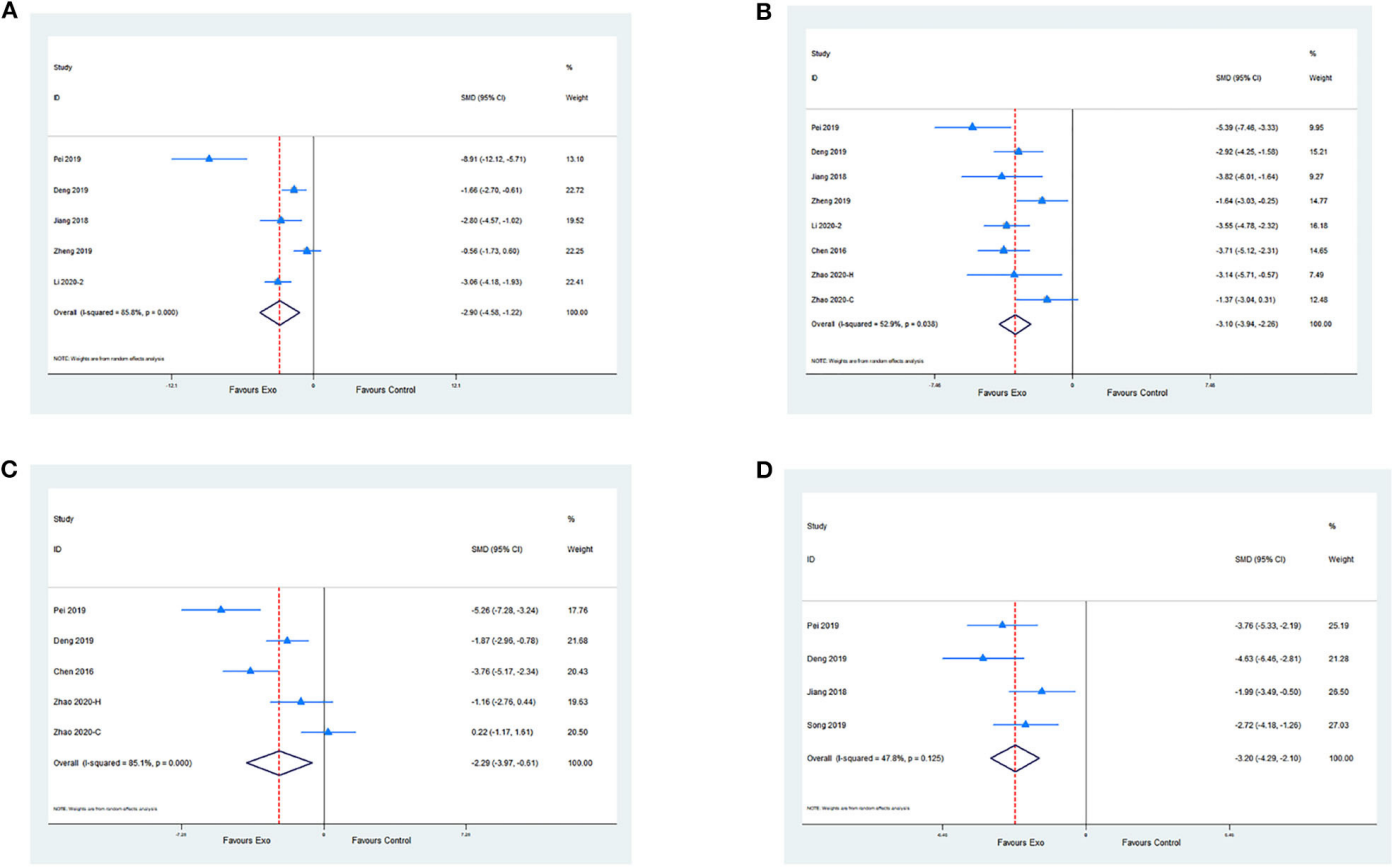
**(A)** Pooled estimate of exosomes on anti-inflammation, according to IL-6. **(B)** Pooled estimate of exosomes on anti-inflammation, according to TNF-α. **(C)** Pooled estimate of exosomes on anti-inflammation according to IL-1β. **(D)** Pooled estimate of exosomes on anti-apoptosis according to terminal deoxynucleotidyl transferase deoxyuridine triphosphate nick end labeling-positive cells.

Five studies evaluated the anti-inflammatory effect of exosomes by measuring IL-6 levels compared with the control. Meta-analysis presented a significant reduction in the exosome group (SMD −2.9; 95% CI −4.58 to −1.22; *p* < 0.001; *I*^2^ = 85.8%; Figure 4A). A meta-analysis of seven studies with eight comparisons indicated that the level of TNF-α was significantly decreased in the exosome group compared with the control group (SMD −3.10; 95% CI −3.94 to −2.26; *p* < 0.05; *I*^2^ = 52.9%; Figure 4B). Moreover, four studies with five comparisons assessed the IL-1β level. Meta-analysis showed a significant decrease in the exosome treatment group (SMD −2.29; 95% CI −3.97 to −0.61; *p* < 0.001; *I*^2^ = 85.1%; Figure 4C). A meta-analysis of four studies showed a significant reduction of terminal deoxynucleotidyl transferase deoxyuridine triphosphate nick end labeling-positive cell percentage between the exosome group and the control group (SMD −3.20; 95% CI −4.29 to −2.1; *I*^2^ = 47.8%; *p* = 0.125; Figure 4D; Supplementary Table 2).

### Publication Bias

**Figure 5:**
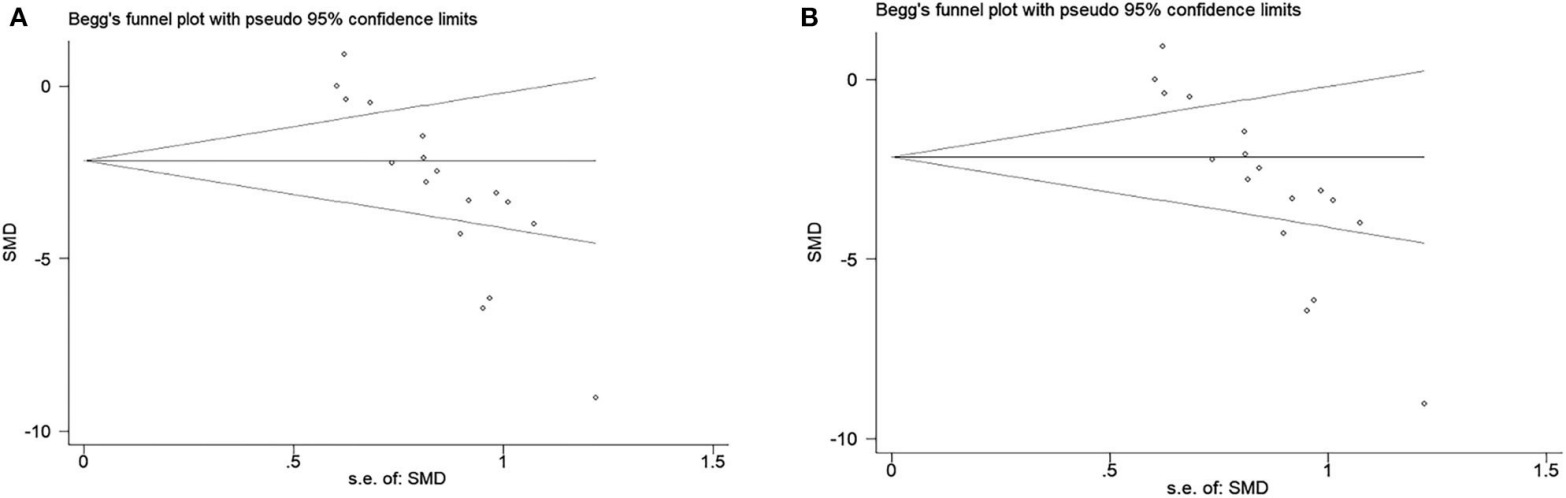
**(A)** Beggs funnel plot with neural function score. **(B)** Beggs funnel plot with infarct volume.

The funnel plot was conducted to examine publication bias for the primary outcomes. Potential publication bias of primary outcomes was tested using Egger's test (*p* < 0.0001; Figures 5A,B). The publication bias was more likely to generate from different types of exosome applications or inconsistent measurements. Hence, we performed several subgroup analyses to figure out the potential sources of publication bias, which are described above in primary outcomes.

## Discussion

To the best of our knowledge, this is the first meta-analysis to systematically describe ischemic stroke with unmodified cell-derived exosomes treatment. A meta-analysis of 18 studies with 21 comparisons presented a comprehensive summary of the pooled effect of cell-derived exosomes on rodents with ischemic stroke. The available evidence from our included studies indicated that stem cell-derived exosomes substantially improved neurological function and reduced IV in a rodent with cerebral ischemia. Moreover, pooled analyses demonstrated that exosome therapy not only ameliorates the inflammatory response but also reduces cell apoptosis after ischemic stroke. Therefore, our meta-analysis provided crucial clues for human clinical trials on exosomes and ischemic stroke.

A previous meta-analysis evaluated the efficacy of extracellular vesicles therapy for stroke (Thomas et al., 2020). Both modified and unmodified cell-derived extracellular vesicles were included to explore their benefits in ischemic and hemorrhagic stroke in this publication, whereas it was tough to identify the real therapeutic effects induced by extracellular vesicles or signaling molecule added artificially. In contrast, we specifically investigated the effects of unmodified cell-derived exosomes on an ischemic stroke to reduce the sources of heterogeneity and improve the overall quality of the included studies. Moreover, our meta-analysis further evaluated the effect of exosomes on pro-inflammatory cytokines and cell apoptosis, providing useful information for further laboratory studies and clinical trials.

In the current study, the subgroup analysis showed that all administrational routes helped improve the neurological function, except for internal jugular vein administration. The result that internal jugular vein administration is not effective might be relevant to the short interval between exosome intervention (2 h) and functional assessment (24 h) and/or the relative insensitivity of a four-point test using neurologic deficit score. Besides, the organ sequestration of circulating exosomes injected intravenously or inadequate dosing may be contributed to this negative result (Sun et al., 2019). In this meta-analysis, 12 included studies applied to the rats, whereas nine studies used mice. The subgroup analysis showed that either mice or rats have significant and positive effects on IVs and neurological scores (Supplementary Figure 3), but the effects of exosomes on IV between-group difference did reach statistical significance (QB = 7.38, *P* = 0.007), which demonstrated in terms of reducing IVs, a larger treatment effect of exosomes on rats than mice.

Remarkably, all studies were performed on male rodents. Indeed, hormone and sex hormone-independent mechanisms played a central role in regulating sex differences in stroke (Wilson, 2013). Immune response, a part of sex hormone-independent mechanisms, is a vital factor in determining the difference for males and females to respond to experimental stroke (Dotson and Offner, 2017). A study reported by Dotson et al. revealed that splenectomy before middle cerebral artery occlusion significantly decreased the peripheral macrophages/monocytes and activated T cells in male C57BL/6J mice but not female, which resulted in attenuation of neurological impairment in male mice but not female (Dotson et al., 2015).

Exosomes, endosome-derived nanosized membrane vesicles, are released by most cell types. Moreover, exosomes are vital carriers for regulating angiogenesis, neurogenesis, inflammation, and cell apoptosis (Deng et al., 2019; Tian et al., 2019; Ling et al., 2020; Zhao et al., 2020). Emerging studies demonstrated that stem cell transplantation improved neurological recovery after ischemic stroke, and a previous meta-analysis showed that stem cell therapy was safe and effective in animals and humans with cerebral ischemia (Xin et al., 2013a). More importantly, recent studies reported no significant difference in efficacy between stem cell treatment and stem cell-derived exosomes in a rodent model with ischemic stroke. Stem cell-derived exosomes take part in cell-to-cell communication and are considered as the paracrine effectors of cell-based therapy by transferring a great deal of signaling factors including proteins, lipids, mRNA, and non-coding RNAs (such as miRNAs) (Liang et al., 2014; Xin et al., 2014). Compared with cell-based treatment, cell-derived exosomes have lower immunogenicity. In addition, exosome administration could reduce or avoid some of the risks related to cell translation, such as tumorigenesis and cytokine release syndrome (Phinney and Pittenger, 2017). Thus, exosomes seem to be a promising therapy for the recovery of ischemic stroke.

Increasing studies have demonstrated that RNAs derived from exosomes are the critical component for their therapeutic effect. As the content of exosomes, miRNAs are ~22 nucleotides in length and function as non-coding RNAs regulating the level of protein expression by modulating mRNA translation (Ha and Kim, 2014). The miRNAs participate in post-transcriptional regulation of gene expressions typically by binding to the 3′-untranslated region of mRNA sequences (Rupaimoole and Slack, 2017). Given the dramatic effects of miRNA, exosomal miRNAs have gained more attention than proteins and lipids. More and more studies demonstrated that the exosomal miRNAs were involved in the processes of neurological recovery and neural remodeling after stroke (Xin et al., 2017a; Zhang et al., 2019).

In our included studies, there are numerous miRNAs correlated with the neuroprotective effects of exosomes, such as miR-17-92 (Xin et al., 2017a), miR-126 (Venkat et al., 2019), miR-124 (Song et al., 2019), miR-26a (Ling et al., 2020), miR-26b-5p (Li G. et al., 2020), miR-30d-5p (Jiang et al., 2018), and miR-138-5p (Deng et al., 2019). For example, miR-17-92 contained in exosomes enhanced neural plasticity and improved neurological function after stroke, possibly via downregulating PTEN expression and subsequent activating its downstream proteins, the PI3K/Akt/mTOR/GSK-3β signaling pathway (Xin et al., 2017a). Song et al. (2019) demonstrated that M2 microglia-derived exosomes alleviated ischemia-reperfusion injury and protected neuronal survival, and the underlying mechanism might be relevant to exosomal miR-124 and its downstream target ubiquitin-specific protease 14. Moreover, a study reported by Venkat et al. (2019) demonstrated that endothelial cells-Exo promoted capillary tube formation and improved axonal remodeling. However, the downregulation of miR-126 in endothelial cells-Exo reduced Exo-afforded effects of capillary tube formation and axonal regeneration. Thus, it means that exosomal miRNAs play a key role in the neuroprotective function and neural recovery after stroke.

Inflammation is widely involved in the pathogenesis of numerous cerebrovascular disorders, such as ischemic stroke and intracerebral hemorrhage (Kleinig and Vink, 2009), leading to secondary injury to the brain. Increasing evidence demonstrated that microglia-modulated inflammation actively played a part in cerebral infarction (Cai et al., 2017). When ischemic stroke occurred, microglia were polarized toward the M1 phenotype, which produces multiple pro-inflammatory cytokines, including IL-6, TNF-α, and IL-1β, thus aggravating neurofunctional impairment (Tobin et al., 2014). In contrast, the microglia M2 phenotype promotes the secretion of anti-inflammatory cytokines, such as IL-4 and IL-10, thus alleviating neuroinflammation and improving neuronal survival (Schmieder et al., 2012). Li et al. reported that the pro-inflammatory cytokines TNF-α, IL-1β, and IL-6 in the brain tissue were significantly elevated in the rats with ischemic stroke (Li S. et al., 2020).

In this study, six included studies demonstrated that exosome treatment significantly decreased the production of pro-inflammatory factors in brain tissue after cerebral ischemia (Chen K. H. et al., 2016; Deng et al., 2019; Pei et al., 2019; Zheng et al., 2019; Li G. et al., 2020; Zhao et al., 2020). On the other hand, Zhao et al. found that exosome treatment promoted the secretion of anti-inflammatory cytokines (TGF-β and IL-10) in the ischemic hemisphere and repressed microglia M1 polarization to inhibit microglial inflammation via regulating the CysLT 2R-ERK1/2 pathway (Zhao et al., 2020). Moreover, a study reported by Jiang et al. demonstrated that exosomes secreted from adipose-derived stem cells upregulated the expression of anti-inflammatory cytokines (IL-4 and IL-10) and enhanced M2 microglia/macrophage polarization (Jiang et al., 2018). Therefore, exosomes exert a potential neuroprotective effect partially through attenuating brain inflammation.

The ischemic core and penumbra are two separate vital areas of the brain during ischemic stroke. The ischemic core experiences an extreme and rapid decrease of blood flow with irreversible cell death a few minutes after cerebral ischemia. However, apoptosis within the penumbra may appear after several hours or days with reversible cell death (Bandera et al., 2006). Thus, apoptosis is another essential part of the pathogenesis of ischemic stroke, and inhibition of apoptosis could alleviate cerebral injury in stroke models (Uzdensky, 2019). Our meta-analysis demonstrated that cell-derived exosomes contributed to the reduction of cell apoptosis. Song et al. indicated that treatment with M2 microglia-derived exosomes greatly attenuated apoptotic neurons and protected from ischemia-reperfusion injury after stroke compared with phosphate-buffered saline treatment (Song et al., 2019). Moreover, Pei et al. (2019) demonstrated that astrocyte-derived exosomes significantly decreased middle cerebral artery occlusion that induced neuron apoptosis via inhibiting autophagy in experimental ischemic stroke. Additional research reported by Xin et al. (2017a) demonstrated that exosomes not only promoted neurogenesis and oligodendrogenesis but also improved neuronal dendrite plasticity in the ischemic boundary zone, which is possibly related to the PI3K/Akt/mTOR/GSK-3β signaling pathway. Besides, endothelial cell-derived exosomes significantly increased artery diameter and vessel density in the ischemic boundary zone compared with stroke mice without any treatment (Venkat et al., 2019).

Exosomes are emerging as essential intercellular carriers in exerting neurorestorative effects after stroke (Tian et al., 2018; Song et al., 2019). The present meta-analysis result showed that both immediate and delayed treatments with cell-derived exosomes provide therapeutic benefits. Moreover, almost all cell-derived exosomes are effective in improving neurological recovery. However, there are multiple translational challenges needed to be solved before the clinical application of cell-derived exosomes. Firstly, the approach of isolation and storage may have an impact on the characteristics and quality of exosomes (Maroto et al., 2017). Appropriate methods of isolation and storage need to be standardized. Secondly, it is vital to develop the optimal methods to produce a sufficient quantity of exosomes to meet the clinical requirement. Thirdly, in most studies, the follow-up time was <1 month. Thus, the long-term effects of exosomes are a pivotal challenge that needs to be further explored. Fourthly, dose-response and therapeutic window researches are required before the clinical application.

Our study has several potential limitations. First, the earliest start time of intervention available in large human clinical trials was a median of 45 min after ischemic stroke onset (Saver et al., 2015). However, there were ten animal experiments adopting exosome therapy immediately after brain ischemia, which may lead to the overestimation of exosome intervention effects achievable. Second, the ischemic stroke model was performed on young, healthy male rodents in most of the included studies. Nevertheless, ischemic stroke usually occurs in elderly male or female individuals with several risk factors of cerebrovascular diseases (such as hypertension, hyperlipemia, and diabetes), indicating that the heterogenicity of the post-stroke patient requires more complicated interventions. Third, it is important to realize that scaling the production of exosomes for human clinical trials would be required. The general consensus on exosome isolation, purification, and normalization procedures would overcome the crucial bottlenecks in translational studies. Finally, several limitations for this meta-analysis itself should be considered. On the one hand, the heterogeneity between the included studies cannot be evidently decreased, even if we performed subgroup and sensitivity analyses. On the other hand, we performed the data extraction from graphics using WebPlotDigitizer software, which may alter the original data and affect the results.

## Conclusion

Pooled data from the present meta-analysis demonstrated that cell-derived exosomes improve neurological function and reduce IV in rodent models of ischemic stroke. Furthermore, we found that cell-derived exosomes have potential neuroprotective effects, which were mainly mediated through anti-inflammation and anti-apoptosis. Despite the fact that some factors, such as publication bias and study quality, may affect the validity of positive results, this meta-analysis provides important clues to translate new therapeutic options for cerebral ischemia.

## Data Availability Statement

The original contributions generated for the study are included in the article/Supplementary Materials, further inquiries can be directed to the Corresponding author.

## Author Contributions

MH and ZH carried out the design, collected the data, performed the statistical analyses, and drafted the manuscript. CX and LL assisted with statistical analyses and drafted the manuscript. LC, SC, and TL participated in the collection of data. HZ conceived the design of the study and contributed to draft the manuscript. All authors contributed to the article and approved the submitted version.

## Conflict of Interest

The authors declare that the research was conducted in the absence of any commercial or financial relationships that could be construed as a potential conflict of interest.

## Supplementary Material

Supplementary Figure 1 **(A)** The forest plot shows the efficacy of exosomes via different administrational time in improving the neurological function in the ischemic stroke model. **(B)** The forest plot shows the efficacy of exosomes to different types of stroke in improving the neurological function in the ischemic stroke model. **(C)** The sensitivity analysis of included studies in neurological function scores.

Supplementary Figure 2 **(A)** The forest plot shows the efficacy of exosomes via different administrational time in reducing infarct volume in the ischemic stroke model. **(B)** The forest plot shows the efficacy of exosomes to different types of stroke in reducing infarct volume in the ischemic stroke model. **(C)** The sensitivity analysis of included studies in infarct volume.

Supplementary Figure 3 **(A)** The forest plot shows the efficacy of exosomes in improving the neurological function in rats and mice with ischemic stroke. **(B)** The forest plot shows the efficacy of exosomes in reducing infarct volume in rats and mice with ischemic stroke.

Supplementary Table 1 PRISMA 2009 checklist.

Supplementary Table 2 Meta-analytic results from included studies.
