# Supplementary figures and images for: Effects of Exosomes on Neurological Function Recovery for Ischemic Stroke in Pre-clinical Studies: A Meta-analysis
Source: Front Cell Neurosci. 2020 Nov 26;14:593130. doi: 10.3389/fncel.2020.593130 (PMC7726242; doi:10.3389/fncel.2020.593130)

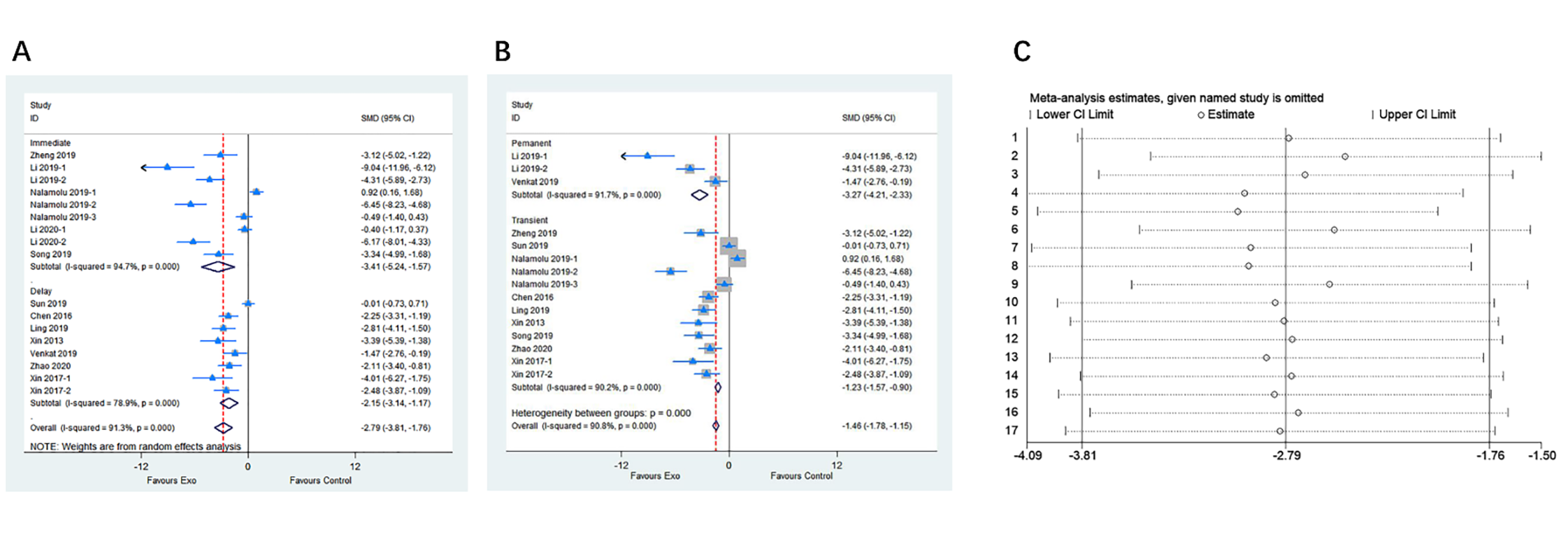

Supplement: Supplementary Figure 1 — (A) The forest plot shows the efficacy of exosomes via different administrational time in improving the neurological function in the ischemic stroke model. (B) The forest plot shows the efficacy of exosomes to different types of stroke in improving the neurological function in the ischemic stroke model. (C) The sensitivity analysis of included studies in neurological function scores. [file Image_1.TIF]

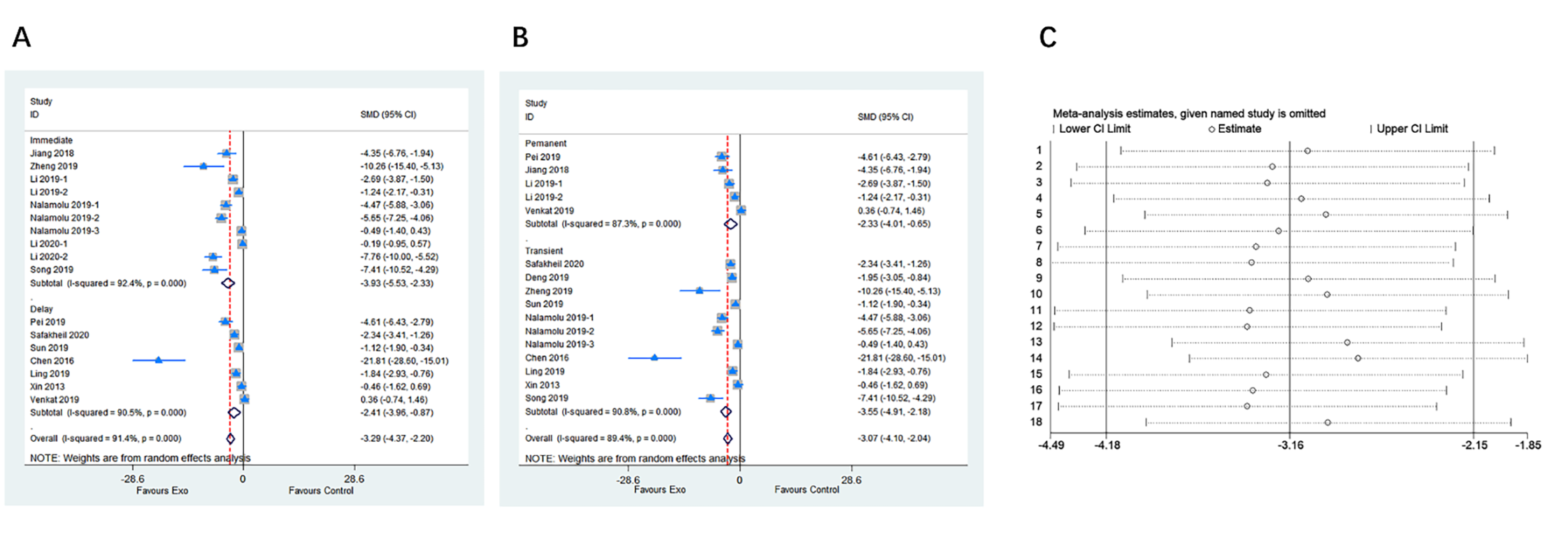

Supplement: Supplementary Figure 2 — (A) The forest plot shows the efficacy of exosomes via different administrational time in reducing infarct volume in the ischemic stroke model. (B) The forest plot shows the efficacy of exosomes to different types of stroke in reducing infarct volume in the ischemic stroke model. (C) The sensitivity analysis of included studies in infarct volume. [file Image_2.TIF]

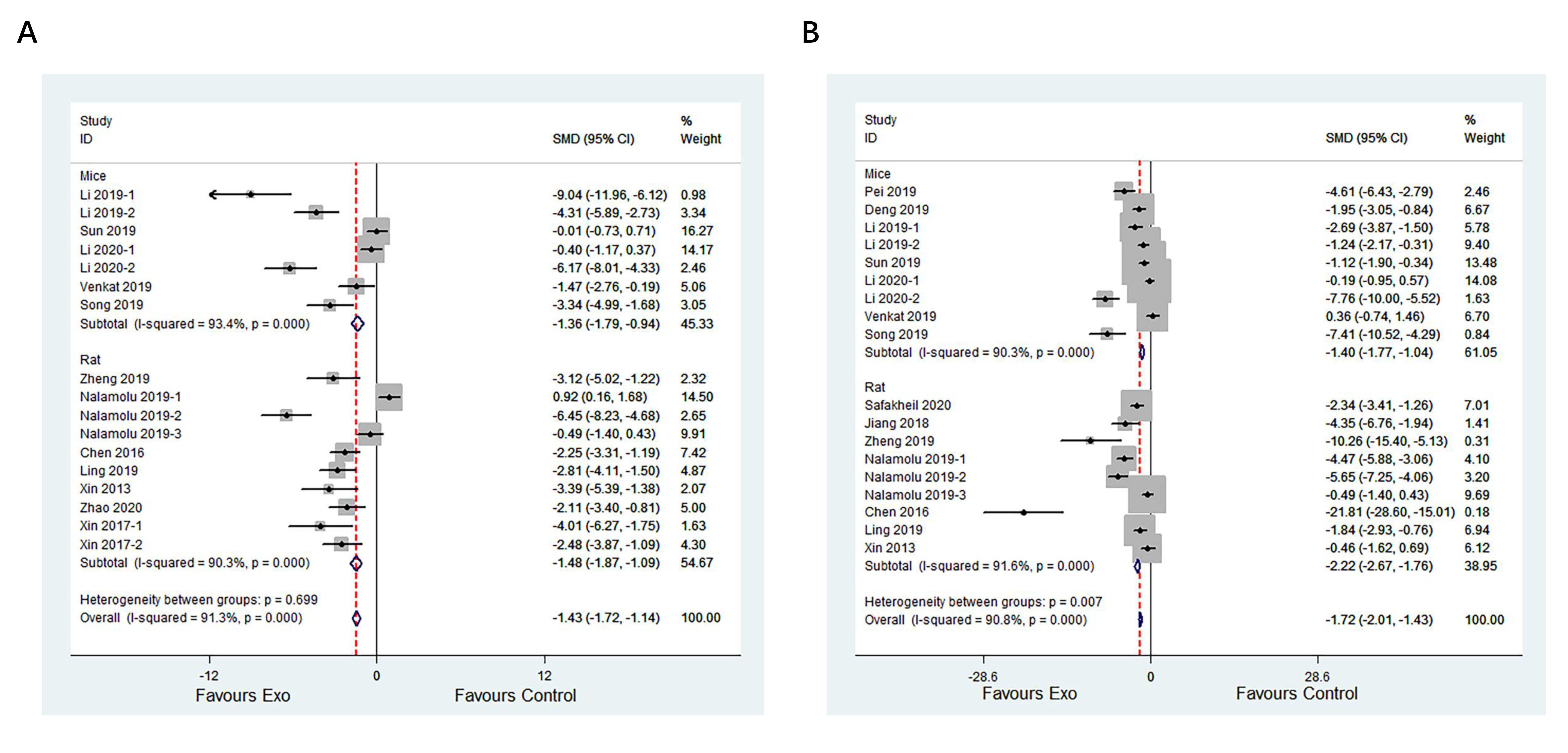

Supplement: Supplementary Figure 3 — (A) The forest plot shows the efficacy of exosomes in improving the neurological function in rats and mice with ischemic stroke. (B) The forest plot shows the efficacy of exosomes in reducing infarct volume in rats and mice with ischemic stroke. [file Image_3.TIF]
