# Supplementary material for: Effects of Exosomes on Neurological Function Recovery for Ischemic Stroke in Pre-clinical Studies: A Meta-analysis
Source: Front Cell Neurosci. 2020 Nov 26;14:593130. doi: 10.3389/fncel.2020.593130 (PMC7726242; doi:10.3389/fncel.2020.593130)
Supplement: Supplementary Table 2 — Meta-analytic results from included studies. [file Table_2.DOCX]

**Table S2** Meta-analytic results from included studies

| **Item** | **N** | **SMD** | **95%CI** | **P**  **value** | ***I^2^*** | **Q**  **P-value** | **QB** | **QB**  **P-value** |
| --- | --- | --- | --- | --- | --- | --- | --- | --- |
| **Primary outcomes** |  |  |  |  |  |  |  |  |
| NFS | 17 | -2.79 | -3.81, -1.76 | < 0.001 | 91.3% | < 0.001 | - | - |
| IV | 17 | -3.16 | -4.18, -2.15 | < 0.001 | 90.8% | < 0.001 | - | - |
| **Secondary outcomes** |  |  |  |  |  |  |  |  |
| IL-6 | 5 | -2.9 | -4.58, -1.22 | < 0.001 | 85.8% | < 0.001 | - | - |
| TNF-a | 8 | -3.10 | -3.94, -2.26 | < 0.001 | 52.9% | 0.038 | - | - |
| IL-1β | 5 | -2.29 | -3.97, -0.61 | < 0.001 | 85.1% | < 0.001 | - | - |
| TUNEL-positive cells | 4 | -3.20 | -4.29, -2.1 | < 0.001 | 47.8% | 0.125 | - | - |
| **Subgroup analyses-NFS** |  |  |  |  |  |  |  |  |
| ***administration site*** |  |  |  |  |  |  | 26.63** | < 0.001 |
| Tail vein | 11 | -1.45 | -1.82, -1.08 | < 0.001 | 93.6% | < 0.001 |  |  |
| IVA | 4 | -2.24 | -2.86, -1.62 | 0.530 | 0.0% | < 0.001 |  |  |
| IAA | 1 | -4.01 | -6.27, -1.75 | - | - | < 0.001 |  |  |
| IJA | 1 | -0.01 | -0.73, 0.71 | - | - | < 0.001 |  |  |
| ***type of ischemia*** |  |  |  |  |  |  |  |  |
| Pemanent | 3 | -3.27 | -4.21, -2.33 | < 0.001 | 91.7% | < 0.001 | 17.26** | < 0.001 |
| Transient | 12 | -1.23 | -1.57, -0.9 | < 0.001 | 90.2% | < 0.001 |  |  |
| ***exosomes source*** |  |  |  |  |  |  | 50.1** | < 0.001 |
| BMSCs | 4 | -2.67 | -3.47, -1.87 | < 0.001 | 0.0% | 0.053 |  |  |
| ADCs | 1 | -2.25 | -3.31, -1.19 | - | - | - |  |  |
| HUCBMSc | 5 | -0.67 | -1.11, -0.24 | < 0.001 | 95.7% | < 0.001 |  |  |
| ECs | 1 | -1.47 | -2.76, -0.19 | - | - | - |  |  |
| MP | 2 | -5.38 | -6.77, -3.99 | 0.005 | 87.2% | 0.06 |  |  |
| GC | 1 | -3.34 | -4.99, -1.68 | - | - | - |  |  |
| Others | 3 | -0.9 | -1.49, -0.30 | < 0.001 | 89.7% | < 0.001 |  |  |
| ***intervention time*** |  |  |  |  |  |  | 0.90 | 0.344 |
| Immediate | 9 | -3.41 | -5.24, -1.57 | < 0.001 | 94.7% | < 0.001 |  |  |
| Delay | 8 | -2.15 | -3.14, -1.17 | < 0.001 | 78.9% | < 0.001 |  |  |
| **Subgroup analyses-IV** |  |  |  |  |  |  |  |  |
| ***administration site*** |  |  |  |  |  |  | 8.63* | 0.035 |
| Tail vein | 12 | -3.64 | -5.04, -2.24 | < 0.001 | 91.7% | < 0.001 |  |  |
| IVA | 3 | -5.12 | -9.56, -0.68 | 0.024 | 95.5% | < 0.001 |  |  |
| Stereotaxic | 1 | -2.34 | -3.41, -1.26 | - | - | - |  |  |
| IJA | 1 | -1.12 | -1.9, -0.34 | - | - | - |  |  |
| ***type of ischemia*** |  |  |  |  |  |  | 0.85 | 0.358 |
| Pemanent | 5 | -2.33 | -4.01, -0.65 | < 0.001 | 87.3% | < 0.001 |  |  |
| Transient | 11 | -3.55 | -4.91, -2.18 | < 0.001 | 90.8% | < 0.001 |  |  |
| ***exosomes source*** |  |  |  |  |  |  | 50.10** | < 0.001 |
| BMSCs | 3 | -1.63 | -2.27, -0.99 | < 0.001 | 66.0% | 0.053 |  |  |
| ADCs | 2 | -6.30 | -8.56, -4.03 | < 0.001 | 95.6% | < 0.001 |  |  |
| HUCBMSc | 5 | -1.73 | -2.23, -1.23 | < 0.001 | 95.5% | < 0.001 |  |  |
| ECs | 1 | 0.36 | -0.74, 1.46 | - | - | - |  |  |
| MP | 2 | -1.79 | -2.52, -1.06 | < 0.001 | 71.7% | 0.060 |  |  |
| GC | 2 | -5.32 | -6.89, -3.75 | < 0.001 | 56.7% | 0.129 |  |  |
| Others | 3 | -1.50 | -2.13, -0.87 | < 0.001 | 84.0% | 0.002 |  |  |
| ***intervention time*** |  |  |  |  |  |  | 3.94* | 0.047 |
| Immediate | 10 | -3.93 | -5.53, -2.33 | < 0.001 | 92.4% | < 0.001 |  |  |
| Delay | 7 | -2.41 | -3.96, -0.87 | < 0.001 | 90.5% | 0.002 |  |  |

*p < 0.05; **p < 0.01; CI = Confidence interval; NFS= Neurological function scores；IV= Infarct volume；IVA= Intravenous administration；IAA= Intra-arterial administration；IJA= Internal jugular vein administration；BMSCs= Bone marrow mesenchymal stem cells；ADCs= Adipose-derived stem cells; HUCBMSc=；ECs= Endothelial cells；MP= Mice plasma；GC= Glial cell.
